# Real-time tracking of stem cell viability, proliferation, and differentiation with autonomous bioluminescence imaging

**DOI:** 10.1186/s12915-020-00815-2

**Published:** 2020-07-03

**Authors:** Michael Conway, Tingting Xu, Andrew Kirkpatrick, Steven Ripp, Gary Sayler, Dan Close

**Affiliations:** 1 490 BioTech, Knoxville, TN 37996 USA; 2 Center for Environmental Biotechnology, The University of Tennessee, Knoxville, TN 37996 USA

**Keywords:** Autobioluminescence, Bacterial luciferase, Bioimaging, *Lux*, Stem cells, iPSC, MSC, Luciferase, Luciferin

## Abstract

**Background:**

Luminescent reporter proteins are vital tools for visualizing cells and cellular activity. Among the current toolbox of bioluminescent systems, only bacterial luciferase has genetically defined luciferase and luciferin synthesis pathways that are functional at the mammalian cell temperature optimum of 37 °C and have the potential for in vivo applications. However, this system is not functional in all cell types, including stem cells, where the ability to monitor continuously and in real-time cellular processes such as differentiation and proliferation would be particularly advantageous.

**Results:**

We report that artificial subdivision of the bacterial luciferin and luciferase pathway subcomponents enables continuous or inducible bioluminescence in pluripotent and mesenchymal stem cells when the luciferin pathway is overexpressed with a 20–30:1 ratio. Ratio-based expression is demonstrated to have minimal effects on phenotype or differentiation while enabling autonomous bioluminescence without requiring external excitation. We used this method to assay the proliferation, viability, and toxicology responses of iPSCs and showed that these assays are comparable in their performance to established colorimetric assays. Furthermore, we used the continuous luminescence to track stem cell progeny post-differentiation. Finally, we show that tissue-specific promoters can be used to report cell fate with this system.

**Conclusions:**

Our findings expand the utility of bacterial luciferase and provide a new tool for stem cell research by providing a method to easily enable continuous, non-invasive bioluminescent monitoring in pluripotent cells.

## Background

Bioluminescence is a powerful tool for visualizing cells and monitoring their physiology. Multiple luciferase reporter systems are available, with firefly (*luc*), Nanoluc (N*luc*), and *Renilla* (R*luc*) luciferase the most commonly employed. These systems have been used, either alone or in combination, to monitor viability [1], gene expression [2], infection progression [3], and a multitude of other applications [4]. The use of luciferases is especially prevalent in mammalian systems, where autofluorescence resulting from requisite excitation wavelength stimulation diminishes signal-to-noise values and complicates data acquisition. However, similar to the required photonic excitation of fluorescent systems, the most widely used luciferases must be activated through the exogenous application of a chemical substrate (luciferin).

Luciferin application can be problematic from economical, logistical, and biological perspectives. The chemical itself is expensive; decomposes upon exposure to light, oxygen, and moisture; and requires frozen storage. In most cases, its application also requires the destruction of the cells under study for luciferase exposure. Biological processing results in dynamic uptake and clearance rates between experiments [5, 6], and chemical interaction produces artifacts in high-throughput operations [7]. These restrictions limit the functionality of luciferases when working with precious samples or those that are destined for further experimentation.

To avoid the complications of external substrate application, two bioluminescent systems have been elucidated that genetically encode both the luciferase and luciferin components required for autonomous functionality: fungal and bacterial luciferases. Fungal luciferase (*luz*) requires the luciferin 3-hydroxyhispidin and the co-factor O_2_. While the oxygen co-factor is readily available in mammalian cells, 3-hydroxyhispidin can be produced from naturally occurring caffeic acid via the expression of 4′-phosphopantetheinyl transferase, hispidin-3-hydroxylase, and a polyketide synthase. This system functions in mammalian cells when exogenously supplemented with luciferin, but genetically encoded luciferin synthesis to support continuous or real-time monitoring has not been demonstrated in these hosts [8].

Bacterial luciferase (*lux*) utilizes fatty aldehyde as luciferin and O_2_ and FMNH_2_ as co-factors. It is thermostable up to 42 °C [9] but has been limited primarily to prokaryotic or single-cellular eukaryotic hosts [10]. Although it functions in mammalian cells [11, 12], it displays reduced luminescent output relative to bioluminescent systems requiring external substrate application and has a peak output at 490 nm, which is relatively blue-shifted compared to the in vivo imaging optimum [13]. The system is also more complex than externally stimulated systems. It is comprised of five genes (*luxCDABE*) that work in a coordinated fashion to express the luciferase heterodimer (consisting of the LuxA and LuxB proteins) and the luciferin generation pathway (consisting of the LuxC, LuxD, and LuxE proteins) [14]. To function in mammalian hosts, the system must also incorporate a flavin reductase gene (*luxF*) to maintain sufficient levels of FMNH_2_ for continuous luminescent production [11]. In this work, we leverage the inherent complexity of this system to re-engineer its expression such that it functions reliably in stem cells and their progeny and to organize the multiple genes under an orientation that permits genetic encoding of conditional expression such that cells self-regulate the initiation or cessation of bioluminescent signals in response to predetermined events.

## Results

### Overexpression of the luciferin synthesis pathway is required for continuous luminescence in iPSCs

**Fig. 1.**
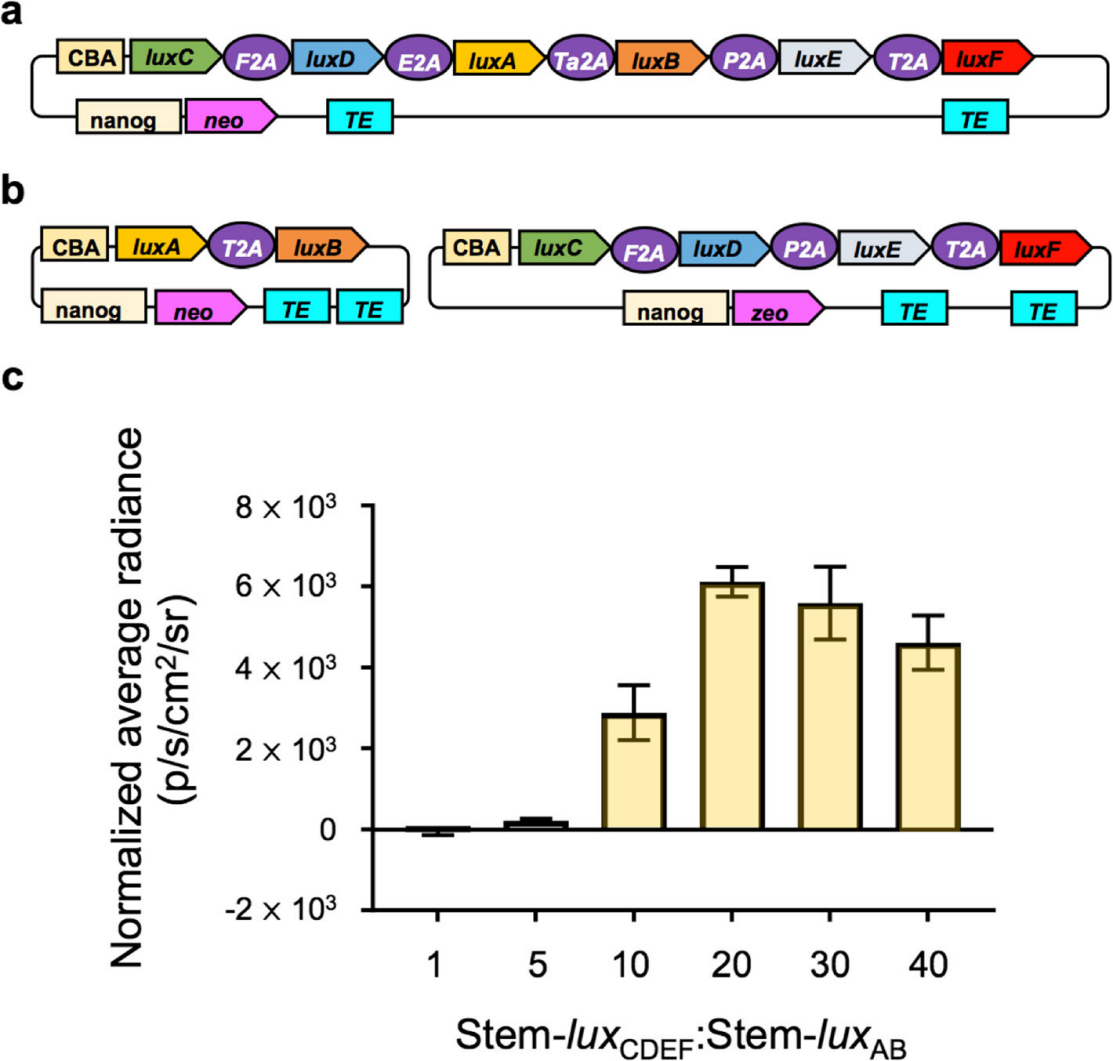
Introducing the *lux* luciferin:luciferase operon components at 20–30:1 M ratios produces robust autobioluminescence in iPSCs. **a** Single operon, 2A-segmented, polycistronic *lux* operon driven by the chicken beta actin (CBA) promoter and flanked by sequence elements facilitating transposon-mediated genomic integration (TE). **b** Split *lux* cassette orientation enabling ratio-based component expression. F2A, foot and mouth disease viral 2A element; E2A, equine rhinitis A viral 2A element; Ta2A, synthetic *Thosea asigna* viral 2A element; P2A, *Porcine teschovirus* 1 viral 2A element; T2A, *Thosea asigna* viral 2A element. **c** Light production following transient transfection of Stem-*lux*_CDEF_:Stem-*lux*_AB_ from **b** at a 1:1 M ratio compared to otherwise identical cells transfected with the same amount of Stem-*lux*_AB_ but increasing molar ratios of Stem-*lux*_CDEF_. The average radiance was normalized to the MTT signal. Values are representative of *N* = 3 replicates. Error bars represent the standard error of the means. p/s/cm^2^/sr; photons/s/cm^2^/steradian. Data is available at https://osf.io/h5qzj/ [15]

The previously published pCMV_*lux*_ vector [12] harbors a synthetic *lux* operon consisting of viral 2A element linked *luxCDABEF* genes under the control of a CMV promoter and has only been shown to function effectively in a handful of immortalized cancer cell lines [11, 12]. pCMV_*lux*_ functionality was confirmed via observation of autobioluminescence following transfection into HEK293 cells (2.09 × 10^5^ (± 4.03 × 10^3^) photons/s) (data is available at https://osf.io/h5qzj/ [15]). To establish a baseline for vector functionality in iPSCs, it was transfected without modification. This approach failed to produce autobioluminescence (20 (± 62) photons/s; *p* = 0.309 compared to untransfected control) (data is available at https://osf.io/h5qzj/ [15]) after transient transfection and following qPCR-based analysis confirming genomic integration of the *luxCDABEF* genes in stably transfected isolates. We thus sought to tailor the *lux* operon for iPSC expression. The CMV promoter can undergo methylation-based silencing in some cell types [16], most notably in embryonic stem cells [17]. Because iPSCs can undergo random methylation dynamics throughout reprogramming and subsequent culture, ultimately resulting in methylation patterns similar to their embryonic stem cell counterparts [18], the viral CMV promoter was replaced with a chicken beta actin (CBA) promoter that provides stable transgene expression in both stem and differentiated cells [19]. This will mitigate any potential promoter silencing while simultaneously improving downstream compatibility in a wider array of differentiated cell types. Similarly, the SV40 promoter driving the neomycin selection marker was replaced with a *nanog* promoter to enable stem cell-specific selection [20] (Fig. 1a). Transfection of this new construct, Stem-*lux*_CDABEF_, resulted in weak but measurable autobioluminescence that did not persist for more than 24–72 h. These observations suggested that *lux* operon expression in iPSCs was capable of supporting autobioluminescence but that some or all of the system components were not expressed sufficiently to support efficient autobioluminescent production.

Viral 2A linker-based expression of multiple open reading frames can result in decreased transcription of the genes distal to the promoter [12]. The operon was therefore divided into its component subsections: the luciferin pathway-encoding *luxCDEF* genes (Stem-*lux*_CDEF_) and the luciferase-encoding *luxAB* genes (Stem-*lux*_AB_) (Fig. 1b). To identify a strategy in which sufficient luciferin would be produced to enable a robust bioluminescent phenotype without negatively effecting host physiology, each component was transiently co-transfected at molar ratios from 1:1 to 40:1 and measuring the resulting light output 24–48 h post-transfection (Fig. 1c). The best performing transient transfection of Stem-*lux*_CDEF_/Stem-*lux*_AB_ resulted in 9.5 × 10^3^ (± 235) photons/s, outperforming the unmodified pCMV_*lux*_ vector under the same conditions (20 (± 62) photons/s) (data is available at https://osf.io/h5qzj/ [15]).

A stable, autobioluminescent iPSC line was generated by co-transfecting the Stem-*lux*_CDEF_ and Stem-*lux*_AB_ vectors using the 20–30:1 M ratio found to produce the brightest signal in the transient transfection experiment, selecting antibiotic-resistant clonal lines, and qualitatively selecting the brightest lineage from among the isolated clones. This line was denoted as iPSC-*lux*. Genomic qPCR-based analysis confirmed the selected lineage had a ~ 27:1 ratio of the luciferin pathway and luciferase genes (Additional file 1: Fig. S1a), which was within the predicted range. Transcriptional analysis showed that despite the 27:1 luciferin:luciferase genomic integration ratio, the luciferin components were transcribed at a ratio of 15.4:1 (± 1.0) (Additional file 1: Fig. S1b). This reduced transcriptional ratio may be due to the positional effects of the insertion location or the difference in the transcribed length of the luciferin generation pathway mRNA compared to the luciferase component mRNA (4470 nucleotides vs 2139 nucleotides).

The growth rate of the iPSC-*lux* line was indistinguishable from that of the wild-type iPSCs (Additional file 2: Fig. S2a), as was its metabolic activity level as measured by ATP content and cell viability as measured by NAD(P)H oxidoreductase activity (Additional file 2: Fig. S2b&c). Long-term culture of this lineage (> 3 months) did not reveal any impact on growth rate relative to the wild-type parent line resulting from the metabolic burden of continuous light production. Autobioluminescent cells retained the expression of pluripotency markers (Additional file 2: Fig. S2d-f) and a normal karyotype (Additional file 2: Fig. S2g&h), suggesting that the integration of the split *lux* operon did not affect the pluripotency or genomic stability.

### Validation of autobioluminescent assays for monitoring viability and proliferation

**Fig. 2.**
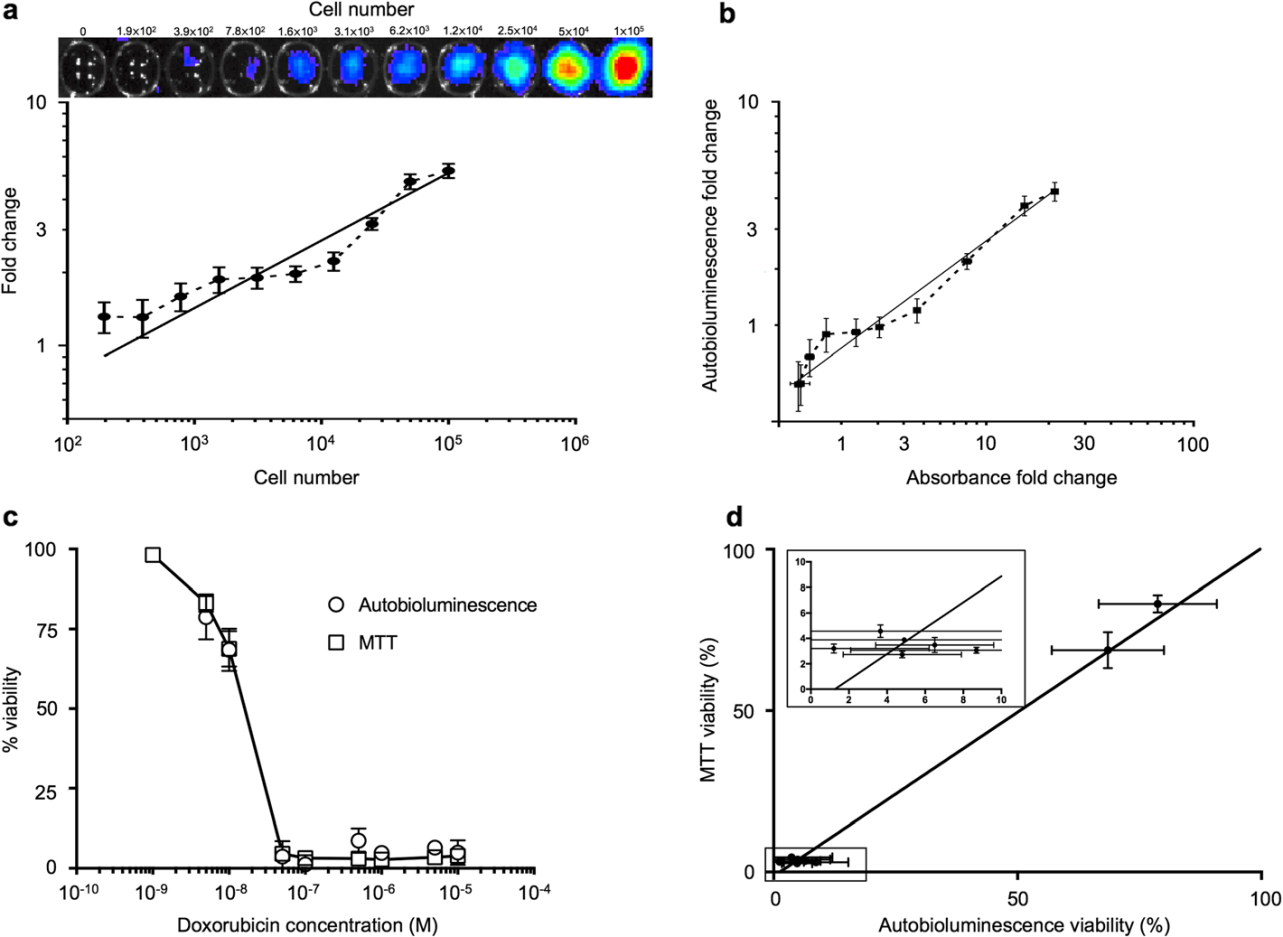
Stem-*lux*_CDEF_/Stem-*lux*_AB_-induced autobioluminescence faithfully recapitulates the results of common assays without necessitating sample destruction. **a** The fold change in autobioluminescence relative to the background correlates strongly with the initial cell seeding density (*R*^2^ = 0.93) to allow a continuous population size determination. *N* = 6 replicates. **b** The fold change in autobioluminescence relative to the background correlates strongly with the fold change in MTT absorbance (570 nm) relative to the background (*R*^2^ = 0.98). *N* = 6 replicates. **c** iPSCs challenged with the indicated doses of doxorubicin report viability without perturbation similarly to the destructive MTT assay. **d** Viability measurements from the two test formats show a strong correlation between the results. The inset shows the correlation between test results under low viability conditions as indicated in the boxed section of the main plot. Values are representative of *N* = 3 replicates. Error bars represent the standard error of the means. p/s/cm^2^/sr; photons/s/cm^2^/steradian. Data is available at https://osf.io/h5qzj/ [15]

If luciferin production is correctly titrated to support consistent enzymatic turnover without modification of host physiology, the continuous, self-generated bioluminescence of the iPSC-*lux* line should correlate linearly with the population size by exhibiting light output proportional to the cell number. To test this, a range of iPSC-*lux* cells were examined for autobioluminescent output. The observed average radiance correlated strongly (*R*^2^ = 0.93) to the number of plated cells (Fig. 2a). These results were validated against MTT assays across all population sizes. Results from the two assay formats were strongly correlated (*R*^2^ = 0.98) (Fig. 2b), suggesting the autobioluminescent assay format is faithfully reporting viable population size. Unlike the colorimetric MTT assay, which uses the NAD(P)H-dependent cellular oxidoreductase enzymes of lysed cells to reduce a tetrazolium dye, using autobioluminescence to assay population size and viability does not require interaction with or destruction of the sample. This avoids imparting unintended influence over the cells and enables continuous observation or further downstream testing post-interrogation.

We next sought to validate whether autobioluminescence could similarly report viability changes in response to toxicological challenge. Treatment of the iPSC-*lux* line with a range of doxorubicin concentrations resulted in dose-dependent changes to autobioluminescent output indicative of changing cellular viability. These results were consistent with validation measurements made using complementary MTT assays (*R*^2^ = 0.99) and yielded similar half-maximal inhibitory concentration (IC_50_) values (autobioluminescence = 1.31 × 10^−8^ M; MTT = 1.41 × 10^−8^ M (Fig. 2c, d). These data illustrate that the autobioluminescent assay is capable of reporting changes in cellular viability resulting from autonomous modulation of the cells’ autobioluminescent output in response to cell stress and death.

### Continuous light production enables lineage tracking post-differentiation

**Fig. 3.**
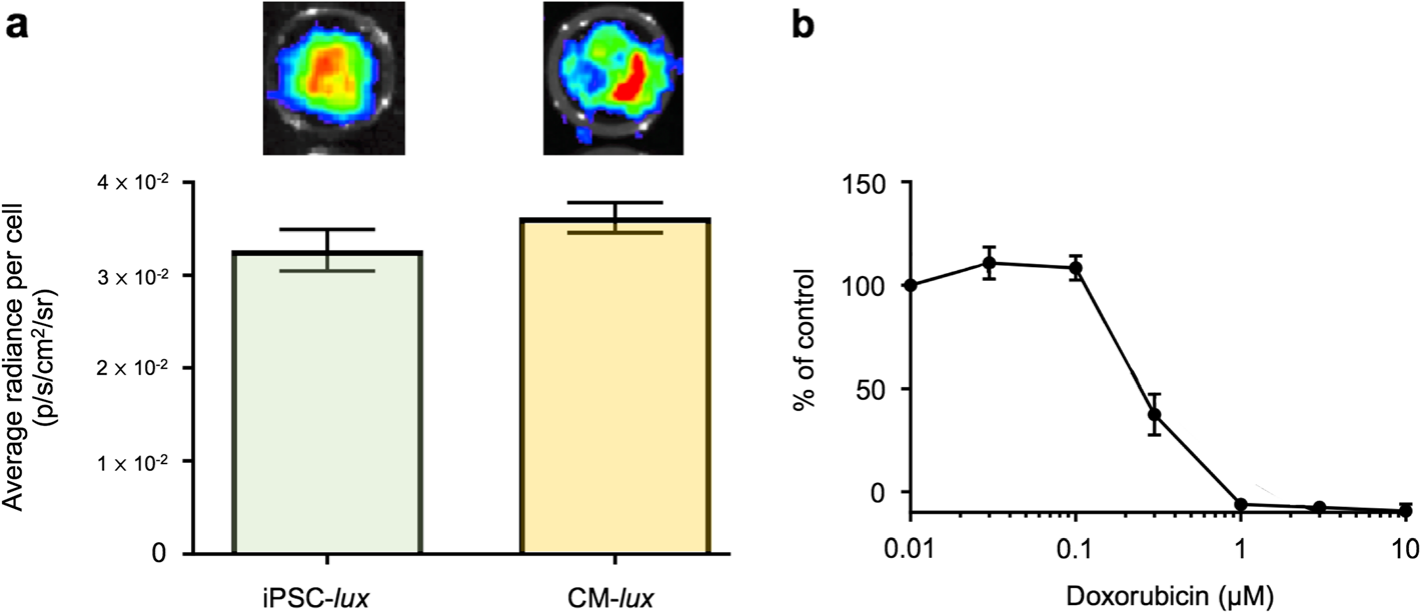
Cardiomyocytes derived from autobioluminescent iPSCs maintain a continuous light production. **a** The autobioluminescent signal from iPSCs with genomically integrated Stem-*lux*_CDEF_*:*Stem-*lux*_AB_ (iPSC-*lux*) is not altered following differentiation into cardiomyocytes (CM-*lux*). **b** Autobioluminescent CM-*lux* cells remain capable of reporting changes in viability in response to doxorubicin challenge and produce IC_50_ values similar to previous reports [22]. Values are representative of *N* = 3 replicates. Error bars represent the standard error of the means. p/s/cm^2^/sr; photons/s/cm^2^/steradian. Data is available at https://osf.io/h5qzj/ [15]

We sought to test whether the autobioluminescent phenotype is preserved in progeny differentiated from the continuously luminescent iPSC-*lux* line. Derivation of iPSCs into cardiomyocytes enables the exploration of cardiotoxicity and cardiac biology. Enabling the continuous assessment of cellular health and metabolic activity across this transition and beyond would be a beneficial research tool. Targeted cardiac differentiation [21] of the iPSC-*lux* line produced autobioluminescent cardiomyocytes**.** The autobioluminescent signal of the derived cardiomyocytes (CM-*lux*) did not differ from that of their iPSC progenitors (Fig. 3a), suggesting the autobioluminescent phenotype is not radically altered by the change in cell type. We next sought to determine whether CM-*lux* cells retained the ability to report cytotoxic exposure impacts via self-modulation of autobioluminescence. Following a 24-h exposure to a range of doxorubicin concentrations, CM-*lux* autobioluminescence decreased in a dose-response fashion (Fig. 3b) with an IC_50_ of 0.29 μM. This value agrees with the published IC_50_ value as calculated using PrestoBlue, an alternative non-lytic assay that uses a resazurin-based solution to measure the reducing power of living cells and been correlated with cardiomyocyte viability, contractility, electrophysiology, calcium handling, and signaling [22].

### Autobioluminescent iPSC-derived models increase per sample data output by transitioning endpoint measurements to kinetic assays without necessitating alterations to existing protocols

Cardiotoxicity testing is an essential part of therapeutic development and non-therapeutic chemical risk assessment. While continuous cell monitoring is possible for these applications (e.g., impedance plates), the equipment and consumable costs for these approaches are significantly high as to inhibit their common application. Thus, these screens more commonly utilize iPSC-derived cardiomyocytes within a destructive end-point assay that yields only a single measurement time point. In this format, orchestrating replicates to capture kinetic toxicity data becomes expensive and introduces experimental variation even with modest increases in scale. To address this problem, we sought to validate the use of CM-*lux* cells to provide real-time, continuous cardiotoxicity monitoring in response to a chemical challenge over an extended time.

**Fig. 4.**
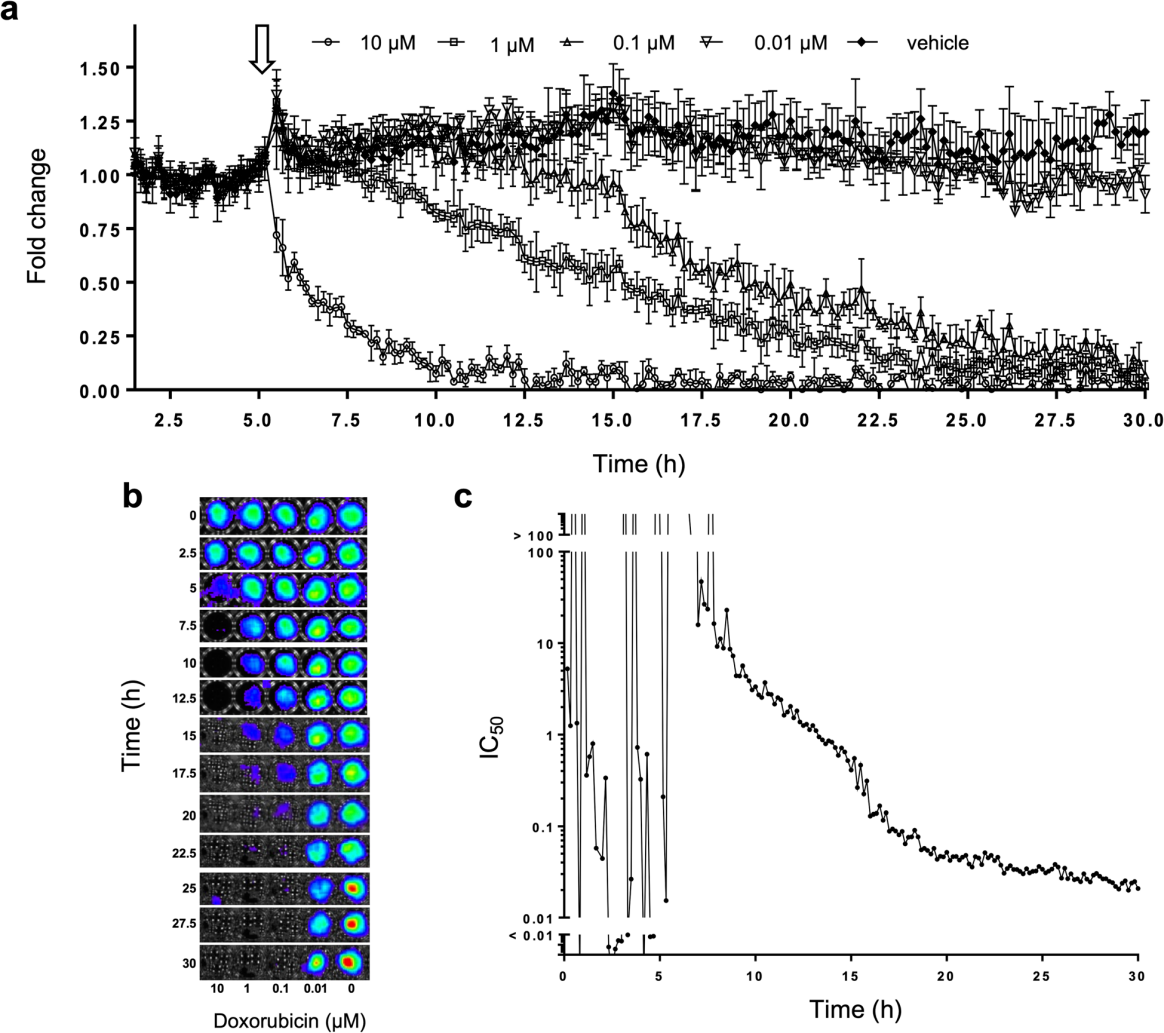
Autobioluminescent cardiomyocytes enable kinetic doxorubicin toxicity monitoring over prolonged time periods. **a** CM-*lux* autobioluminescence pre- and post-challenge with increasing doses of doxorubicin (challenge was introduced at the time indicated by the white arrow). **b** Representative pseudocolor images of CM-*lux* autobioluminescent signal at 2.5-h intervals over the time series shown in **a**. **c** The calculated IC_50_ of doxorubicin plotted against time. Values are representative of *N* = 3 replicates. Error bars represent the standard error of the means. p/s/cm^2^/sr; photons/s/cm^2^/steradian. Data is available at https://osf.io/h5qzj/ [15]

CM-*lux* cells were continuously monitored for 5 h to establish a baseline signal. Subsets of cells were then challenged with a range of doxorubicin concentrations while continuing to measure light output over the next 25 h. Increasing doxorubicin concentrations resulted in decreasing autobioluminescent output across the post-treatment monitoring window (Fig. 4a). The continuous data show that higher concentrations of doxorubicin exert toxic effects faster than lower doses despite the different concentrations resolving to approximately the same level of autobioluminescent output by the end of the assay. This trend remains when examining 2.5-h intervals (Fig. 4b), indicating that the assay can be recapitulated using equipment that is not capable of real-time image acquisition. The calculation of IC_50_ values over the experimental time course revealed a reduction in IC_50_ concentration with time (Fig. 4c). While this is expected for a known cardiotoxic compound like doxorubicin, the kinetic values provide an enhanced context for determining the IC_50_ value by allowing this measurement to be performed after the value stabilizes, thus enabling a more confident assessment of toxicity.

### Real-time transcriptional activation or tissue-specific differentiation bioreporters can be easily produced by regulating luciferase component expression with appropriate promoters

**Fig. 5.**
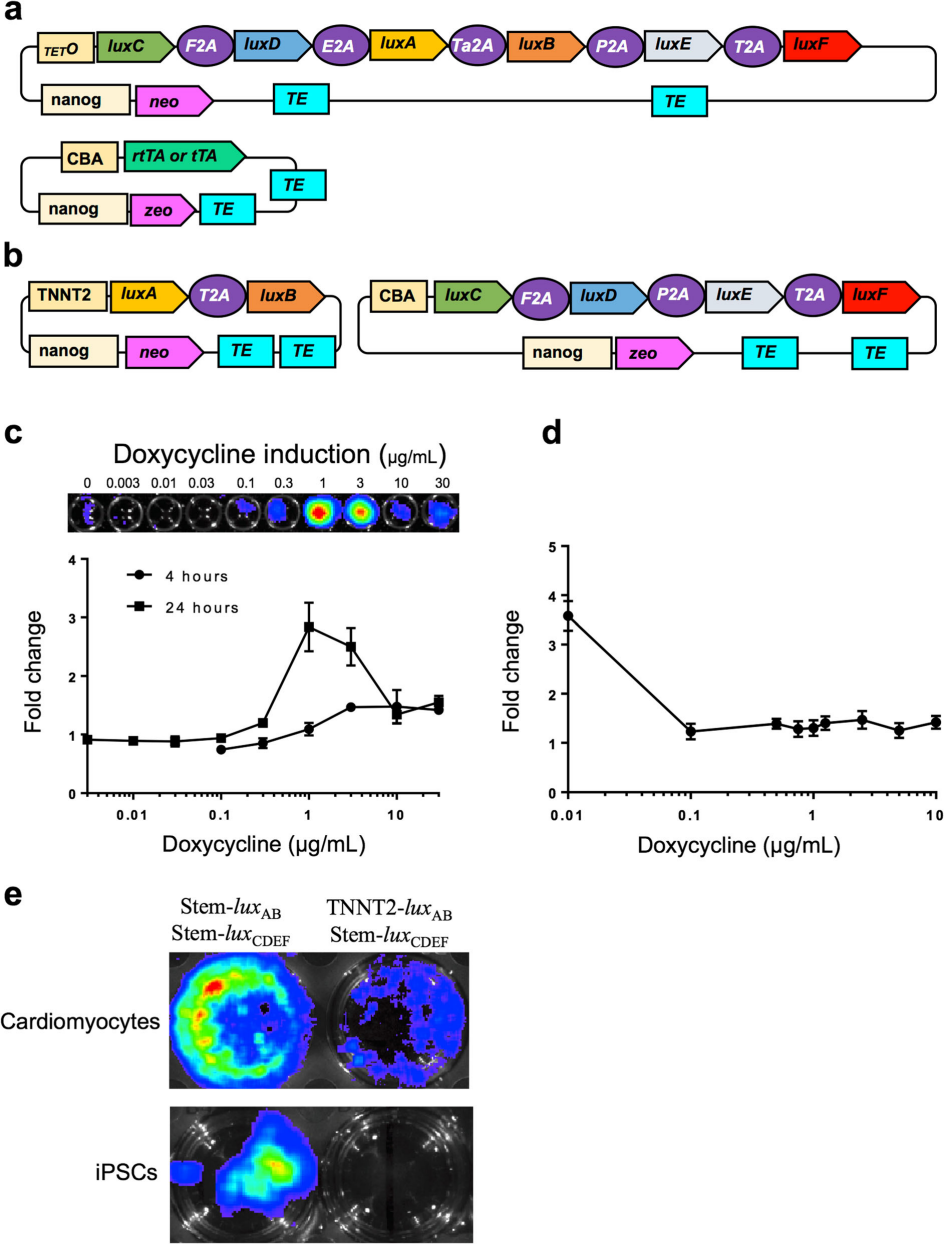
Autonomous reporting of transcriptional activity and tissue identification using autobioluminescence. **a** Two vector inducible or repressible autobioluminescence cassette schematics. The first vector uses a modified tetracycline response element (_TET_O) to control the expression of the viral 2A-segmented, polycistronic *lux* operon. In the second vector, CBA drives the expression of either a transactivator (tTA) that provides constitutive *lux* expression until it is repressed in the presence of doxycycline or a reverse transactivator (rtTA) that inhibits *lux* expression until doxycycline is present. **b** For tissue-specific reporting, the luciferase component genes are controlled by the cardiac-specific TNNT2 promoter. **c** iPSCs with the *lux* genes under the control of the tetracycline responsive promoter and a separately integrated CBA-driven reverse transactivator (rtTA) produce autobioluminescence when exposed to increasing amounts of doxycycline for either 4 or 24 h. **d** In contrast, iPSCs with the *lux* genes under the control of the tetracycline responsive promoter and a separately integrated CBA-driven transactivator (tTA) show a reduction in autobioluminescent output in response to doxycycline exposure. Values are representative of *N* = 3 replicates. Error bars represent the standard error of the means. p/s/cm^2^/sr; photons/s/cm^2^/steradian. **e** Both wild-type iPSCs and iPSC-derived cardiomyocytes produce autobioluminescence when transfected with Stem-*lux*_CDEF_/Stem-*lux*_AB_, but only cardiomyocytes produce light when the Stem-*lux*_CDEF_/TNNT2-*lux*_AB_ vectors are used. Data is available at https://osf.io/h5qzj/ [15]

To expand the utility of this approach, we sought to test whether autobioluminescence could be controlled with an inducible promoter to report changes in gene expression. To determine if this was possible using a model system, an improved tetracycline promoter was cloned upstream of the luciferase and co-integrated with either a control vector expressing a tTA transactivator (doxycycline exposure activates binding of the transactivator to the TET operator and inhibits transcription of the luciferase genes) or an rtTA reverse transactivator (doxycycline exposure inhibits binding of the transactivator to the TET operator and allows transcription of the luciferase genes) [23] (Fig. 5a). This allowed interrogation of inducible and repressible expression using the highly characterized tetracycline transactivator system. Both the inducible and repressible iPSC lines were capable of self-modulating autobioluminescent expression in response to doxycycline exposure (Fig. 5c, d) and retained their ability to report cardiotoxicity when differentiated into cardiomyocytes (Additional file 3: Fig. S3). To determine if the inducible expression approach could be leveraged to achieve tissue-specific functionality, the cardiac tissue-specific TNNT2 promoter [24] was cloned into Stem-*lux*_AB_ in place of the CBA promoter to create TNNT2-*lux*_AB_ (Fig. 5b) and co-expressed with Stem-*lux*_CDEF_ in iPSCs and iPSC-derived cardiomyocytes. Autobioluminescent expression was observed only in the cardiac cells (Fig. 5e). These results demonstrate transcriptional activation monitoring without necessitating external stimulation to allow for automated signal tracking under normal growth conditions. Using this approach, specific cellular lineages can be endowed with a persistent autobioluminescent phenotype that enables longitudinal proliferation and viability tracking.

### Ratio-based luciferin:luciferase expression similarly enables continuous bioluminescence in MSCs

Having established autobioluminescence in iPSCs and iPSC-derived cardiomyocytes, we reasoned that multipotent mesenchymal stem cells should also be capable of autobioluminescent light production using this approach. To test this, we established an optimal Stem-*lux*_CDEF_:Stem-*lux*_AB_ expression ratio through the transfection of human adipose-derived mesenchymal stem cells (hADMSC) (Additional file 4: Fig. S4a). This ratio was identified to be identical to that for iPSCs, between 20 and 30:1. Autobioluminescence from MSCs expressing Stem-*lux*_CDEF_ and Stem-*lux*_AB_ strongly correlated with cell number (Additional file 4: Fig. S4b) and could be imaged in vivo following intraperitoneal (IP) injection into a mouse model (Additional file 5: Fig. S5a). Autobioluminescent signals from injected cells correlated strongly with cell number (*R*^2^ = 0.99) (Additional file 5: Fig. S5b), suggesting this approach can be used to non-invasively monitor changes in cell population sizes for applications such as tissue regeneration or tumor formation/treatment. To demonstrate that autobioluminescent MSCs can be used to track cell migration, cells were injected into the tail vein and allowed to circulate for 1 h. At this time, specific accumulation in the lungs was readily detectable (Additional file 6: Fig. S6). These findings are consistent with previous reports of MSC accumulation under this experimental design [25].

## Discussion

Bioluminescent assays are routinely used to localize and monitor cells in vitro and in vivo. However, the luciferases commonly employed in these assays necessitate the destruction of the cells under study and result in only intermittent snapshots of data. These limitations stem from the requirement to add an exogenous chemical luciferin. Because the applied luciferin is finite, it is only functional until oxidation during the bioluminescent reaction. This limits data collection to a single point. Furthermore, applied luciferin bioavailability is dynamic over time due to the constantly changing amount of unprocessed luciferin, the physiology of the cell at the time of application, and the route of administration [5, 6]. Nonetheless, the high signal-to-noise ratio of bioluminescent reporters relative to their fluorescent counterparts and the availability of different luciferin variants that can modulate the kinetics of the bioluminescent reaction [26, 27] make bioluminescence a preferred imaging modality despite its disadvantages. Developing alternative bioluminescent systems, such as the bacterial luciferase system, that obviate these hurdles further improves the utility of bioluminescence in vitro and in vivo.

The primary concern surrounding bacterial luciferase gene cassette expression in mammalian cells is the potential toxicity of the fatty aldehyde luciferin [28]. This is especially true when the luciferase genes are modulated to enable autonomous reporter functionality while luciferin synthesis occurs constitutively. Previous reports suggest that 1:1 luciferin:luciferase expression ratios do not produce sufficient fatty aldehyde to exert negative biochemical or phenotypical effects in human cells [11, 12]. In this work, the luciferin synthesis genes are expressed 27:1 relative to the luciferase genes (Additional file 1: Fig. S1). None of the tested cells displays abnormal growth rates or other phenotypic changes (Additional file 2: Fig. S2). However, the transfection of luciferin:luciferase gene ratios below 20:1 and beyond 30:1 shows decreased autobioluminescent output (Fig. 1c). Given that the transcriptional expression ratio of the luciferin:luciferase genes following stable selection was found to be similar to their transfected molar ratio (Additional file 1: Fig. S1), these results suggest that lower luciferin pathway transcriptional ratios are likely insufficient to support substrate generation at a level capable of supporting robust light production, while higher ratios likely result in fatty aldehyde levels that interfere with cellular metabolism and therefore reduce light output. The 20–30:1 ratio appears to be an ideal balance for providing sufficient luciferin to drive the reaction forward while avoiding complicating effects.

It has been suggested that intentionally limiting substrate production can decrease luminescent output relative to externally supplemented systems, such as firefly luciferase, that saturate the luciferase with exogenous luciferin [13]. Alternative bacterial luciferase coding sequences with increased luminescence have been published [1, 29], and it has recently been shown that improved codon optimization strategies can significantly improve luminescence to be similar to firefly luciferase while maintaining lower luciferin:luciferase ratios than those identified as optimal for stem cell expression [30]. To facilitate comparisons between *lux* cassette expression strategies and elucidate the baseline functionality of bacterial luciferase in stem cells, this work utilized the codon usage pattern and viral 2A linker region orientations from the first demonstration of bacterial luciferase in mammalian cells [11]. This format is the most widely available version of the mammalian-optimized bacterial luciferase cassette, and therefore, our results should represent what could be expected if the system is deployed with minimal modification.

The previous bacterial luciferase system only displayed efficient signal expression in a handful of immortalized cancer cell lines [11, 12] and was not capable of producing any signal greater than the background when transfected into iPSCs (this work). The approach detailed in this effort overcomes this lack of functionality. While it is likely that the modification of the promoters used and the adoption of the luciferin pathway overexpression strategy both contributed to enabling stem cell-based autobioluminescent production, we hypothesize that the overexpression strategy provided the largest impact on functionality. The original CMV promoter is a strong promoter and, although potentially subject to silencing in stem cells [17], has been successfully used for iPSC-based transgene expression [31]. We also show that, when using the luciferin pathway overexpression strategy, signal is produced from tissue-specific promoters such as TNNT2 or inducible systems such as the tetracycline transactivator system (Fig. 5). The application of this approach improves the functionality of the bacterial luciferase system by allowing it to function in a wider breadth of cell types than the previous incarnation. Further modification to incorporate the output signal enhancement strategies demonstrated in alternative cell types should further improve the sensitivity and signal-to-noise ratio of bacterial luciferase in stem cells and derived progeny, but may require cell type-specific codon optimization since the reason behind the unusually high impact of codon optimization on expression is not well understood [30].

Although the level of bioluminescent output observed using the least enhanced version of bacterial luciferase was not found to be limiting for in vitro stem cell applications, the relatively low output flux and 490 nm emission maximum of this system increased the difficulty of in vivo signal acquisition. This was overcome by leveraging the consistent, continuous output of the autobioluminescent phenotype to increase photon-counting integration time and capture sufficient signal to distinguish from the background when working with small animal subjects. Despite the extended integration time being shorter than the combined time required to perform luciferin injection, wait for substrate uptake, and perform photon counting using a firefly luciferase reporter, this may still be problematic for some applications. In these cases, the incorporation of recent bacterial luciferase functional enhancement protocols would be an alternative approach for improving signal-to-noise ratios without necessitating increased integration time.

However, because autobioluminescent stem and stem-derived cells remain physiologically similar to their non-bioluminescent counterparts (Additional file 2: Fig. S2) and in vitro performance was suitable using the unenhanced bacterial luciferase, any published version of mammalian-optimized bacterial luciferase can be used to convert traditional endpoint assays to achieve repeated data acquisition. This is especially useful in discovery-based applications where the timing and duration of treatment effects are not known a priori (Figs. 3b and 4; Additional file 3: Fig. S3). By retaining bioluminescence as the output format, these cells allow higher throughput processing in equipment without automated injection pumps to supply exogenous luciferin and enables the transition from endpoint to kinetic assay formats without necessitating the acquisition of new equipment. This provides an increased informational capacity relative to alternative bioluminescent systems while maintaining the non-destructive, lower operational cost, and amenability to automated high-throughput applications attributes of fluorescent systems.

## Conclusions

Given the utility of autobioluminescent systems to self-direct signal activation and deactivation and the widespread use of bioluminescent systems in stem cell models, the adaptation of the bacterial luciferase system to function in iPSC and MSC lines provides a new tool for interrogation of physiological changes. The non-invasive bioluminescent signal and necessity for an intact, viable cell to permit signal generation holds great potential for multiplexing with the current suite of destructive endpoint assays. The incorporation of this approach alongside existing research tools will expand the capabilities of stem cell-based research and provide a facile means for bioluminescent interrogation of precious samples where the use of destructive bioluminescent approaches is logistically infeasible.

## Methods

### Vector construction

Stem-*lux*_CDABEF_ was derived from pCMV_*lux*_ (490 BioTech) [12] by replacing the SV40 promoter driving neomycin resistance with the *NANOG* promoter [20] and the CMV promoter driving *luxCDABEF* to the CBA promoter. The pCMV_*lux*_ CMV promoter was excised with NheI and MluI, and a synthetic 61-bp fragment containing an AscI site (Integrated DNA Technologies) was inserted using the NEBuilder HiFi DNA Assembly Cloning Kit (HiFi; New England Biolabs). The CBA promoter was ligated into the construct using the existing NheI and newly introduced AscI sites. The SV40 promoter was excised with SfiI and SbfI. The *NANOG* promoter was amplified from human genomic DNA using the forward primer 5′-TCAGGCCTCCAAGGCCGCTGGTTTCAAACTCCTGAC-3′ and the reverse primer 5′-CCTCCTCTTCCTCTATACTAAC-3′. The resulting PCR product was digested with SfiI and SbfI and ligated in place of the removed SV40 promoter. Synthetic DNA fragments containing inverted PiggyBac terminal repeats were then added up- and downstream of the cassette expression region using HiFi DNA Assembly. Sanger sequencing was used to confirm successful assembly.

The Stem-*lux*_AB_ vector was created by excising the *NANOG-NEO-CBA* fragment from Stem-*lux*_CDABEF_ with SfiI and AscI. The *luxAB* gene sequence was amplified via PCR using the forward primer 5′-GCAAAGAATTCGCGGCCGCGGTACCGGCGCGCCGGCCTCCGAAACCATGAAG-3′ and the reverse primer 5′-TGCAGGCCGGCCGGATCCTAGGTATACGCGTGCCCGGATCGATCCTTATCG-3′. The modified Stem-*lux*_CDABEF_ vector was digested with AscI and MluI, and the *luxAB* PCR product was inserted into the linearized vector via HiFi cloning. Following assembly, synthetic DNA fragments containing PiggyBac inverted terminal repeats were added up- and downstream of the *luxAB* expression region using HiFi DNA Assembly. The vector was then Sanger sequenced to confirm successful assembly.

The backbone of the Stem-*lux*_CDEF_ construct was generated by HiFi cloning a 1185-bp synthetic DNA fragment (IDT) containing the *NANOG* promoter, the zeomycin resistance gene, and the bGH poly-A sequence upstream of the CBA promoter. The *luxCDEF* gene sequence was prepared by PCR amplifying *luxCDEF* in two individual sections sharing 25 bp of overlap using the upstream primers 5′-GTCTCATCATTTTGGCAAAGAATTCGCGGCCGCGCCACCATGGGCACCAAGAAG-3′ and 5′-CAGGTGGTCGTTGTCCATAGCAATG-3′ and the downstream primers 5′-CATTGCTATGGACAACGACCACCTG-3′ and 5′-GTTAATTAAAGCTTGTTAACGAATTCGGCGCGCCGCTGGTTCTTTCCGCCTCAG-3′. The backbone, upstream and downstream *luxCDEF* fragments, and flanking PiggyBac inverted terminal repeat regions were then assembled into the final Stem-*lux*_CDEF_ construct using HiFi cloning. Assembly was confirmed by Sanger sequencing.

The tetO promoter-driven *luxCDABEF* vector was generated as previously described [12] except that the *NANOG* promoter was used to drive neomycin resistance as described above. The complementary transactivator (tTA and rtTA) vectors were generated by replacing the *luxCDEF* sequence of Stem-*lux*_CDEF_ with either rtTA or tTA by restriction and ligation at the unique NotI and AscI sites. The inserted rtTA and tTA sequences were PCR amplified using the primers 5′-GGCAAAGAATTCGCGGCCGCATGTCTAGACTGGACAAGAGC-3′, 5′-AGCTTGTTAACGAATTCGGCGCGCCTTACCCGGGGAGCATGTCAAGGTC-3′, and 5′-GGCAAAGAATTCGCGGCCGCATGTCTAGATTAGATAAAAG-3′, 5′-AGCTTGTTAACGAATTCGGCGCGCCCTACCCACCGTACTCGTCAATTC-3′, respectively. Following assembly, synthetic DNA fragments containing PiggyBac inverted terminal repeats were added up- and downstream of the gene expression regions using HiFi DNA Assembly. Each construct was verified by Sanger sequencing.

### Cell culture

Episomally reprogrammed human fibroblast-derived iPSCs (Applied Stem Cell) were cultured in Essential-8 Medium (E8; Thermo Fisher Scientific) on growth factor-reduced Matrigel (Corning)-coated tissue culture-treated cultureware at 37 °C with 5% CO_2_ in a humidified incubator. Every 3–5 days, just prior to colony confluence, cells were dissociated with Accutase (Innovative Cell Technologies), diluted, and replated in E8 containing 10 μM Y27632 dihydrochloride (LC Labs). After 24 h in culture, the medium was changed to E8 without Y27632 dihydrochloride until the subsequent passage. MSCs (a generous gift from Dr. Stacey Stephenson of the University of Tennessee Medical Center) were cultured on uncoated tissue culture-treated plasticware in MesenPRO RS Medium (Thermo Fisher Scientific) and passaged at 75% confluence to 3–5 × 10^3^ cells/cm^2^. Genomic integration of autobioluminescent constructs was achieved by electroporation. G418 and/or neomycin was added to the culture medium 72 h after electroporation and used continuously thereafter. Clonal lineage derivation was achieved either by colony picking or cell dilution and expansion. Positive clones were verified by genomic DNA sequencing and autobioluminescent light output.

### Electroporation

Transient and stable iPSC lines were generated using the NEON Transfection system (Thermo Fisher Scientific) according to the manufacturer’s recommendation. Briefly, iPSCs were harvested with Accutase, washed twice with PBS, and resuspended at a concentration of 2 × 10^7^ cells/mL in 10 or 100 μL of Buffer R containing the desired DNA. Cells were co-electroporated with the target lux vectors and a transposase-expressing vector. Immediately following electroporation, cells were diluted in prewarmed E8 containing Y27632 and plated. MSC and cardiomyocyte electroporations were performed identically, except that cardiomyocytes were recovered in Advanced RPMI (Thermo Fisher Scientific) and MSCs were recovered in MesenPRO RS medium (Thermo Fisher Scientific).

### Transient and stable cell line selection

Following transient transfection, electroporated cells were assayed 24 h post-electroporation. Stably transfected autobioluminescent iPSCs were electroporated with the Stem-*lux*_CDEF_ and Stem-*lux*_AB_ vectors as described. Beginning 48 h post-transfection, selective pressure was applied using 100 μg G418/mL and 1 μg zeocin/mL. Antibiotic-supplemented medium was refreshed every 24 h until individual clonal lines were formed. Each resistant lineage was assayed for light production using an IVIS Lumina imaging system (PerkinElmer) and qualitatively rank-ordered based on their autobioluminescent signal intensity. The lineage producing the greatest signal was denoted as iPSC-*lux* and used for further experimentation.

### Cardiac differentiation and culture

Cardiac differentiation was performed as previously described [32, 33]. Briefly, iPS lines were seeded in 12- or 24-well plates. At 2–3 days post-seeding, the cells were treated with CHIR99021 (Tocris) for 24 h. At 3 days post-seeding, the cells were treated with IWP4 (Tocris) for 24 h. At 7 days post-seeding, the medium was switched to Advanced RPMI (Thermo Fisher Scientific). Beating was observed between days 7 and 14, and differentiation was confirmed by observation of beating and immunohistochemical staining with the cardiac-specific anti-Troponin T antibody (Additional file 7: Fig. S7). Cardiomyocytes were maintained in Advanced RPMI supplemented with GlutaMAX.

### qPCR

Genomic DNA was isolated from pellets containing 1–5 × 10^5^ cells previously frozen at − 80 °C using the Quick DNA Miniprep kit (Zymo Research) following the manufacturer’s instructions. qPCR was performed in technical triplicate on the QuantStudio 3 (Applied Biosystems) using the Ambion Power SYBR Power Green Cells-to-CT Kit (Thermo Fisher Scientific) according to the manufacturer’s instructions. *luxA* was probed with primers 5′-GCTACCACTATCTTTGACGACTC-3′ and 5′-GTCGATGCGTCTGTTAGTATCC-3′. *luxD* was probed with primers 5′-GCCAGCACCATCAACAATATG-3′ and 5′-TCACTTCGTCCTGTTTGACC-3′.

### qRT-PCR

mRNA was isolated from pellets containing 1–5 × 10^5^ cells and prepared for qRT-PCR using the Power SYBR Green Cells-to-CT Kit (Thermo Fisher Scientific) according to the manufacturer’s instructions. qRT-PCR was performed in technical triplicate on the QuantStudio 3 (Applied Biosystems). The use of viral 2A linker regions to join the open reading frames of the luciferase and luciferin operons results in the production of a single mRNA for each operon that is broken into individual proteins during translation when the ribosome encounters the 2A peptide region. This results in the luxA:luxB (luciferase) transcriptional levels always being 1:1 and the luxC:luxD:luxE:frp (luciferin generation pathway) transcriptional levels always being 1:1:1:1. Luciferin transcript abundance was probed with primers 5′-CGAGAACCTGGAAAACAAGC- 3′ and 5′-TTGTCGTCCACGATGTTGAT-3′. Luciferin pathway transcript abundance was probed with primers 5′-TGGTGTTCTGCATCGACTACC-3′ and 5′-CAGGCCGCCGATGTACAC-3′.

### Immunohistochemistry

iPSCs were plated on Matrigel-coated petri dishes with optical glass centers (MatTek) and cultured until sufficiently confluent for passage. Cells were fixed with 4% PFA for 15 min at room temperature, washed twice with PBS, permeabilized with 0.4% Triton X-100 in PBS for 5 min at room temperature, and washed twice with PBS. Fixed and permeabilized cells were then blocked in PBS containing 0.4% Triton X-100 and 5% goat serum overnight at 4 °C. The primary antibody was applied at the specified dilution (Additional file 8: Table S1) in PBS containing 2% goat serum and 0.4% Triton X-100 for 2 h at room temperature or overnight at 4 °C. Cells were then washed 4 times with PBS, and the secondary antibody (Additional file 8: Table S1) was added in PBS containing 0.4% Triton X-100 and 5% goat serum for 2 h at room temperature. Cells were washed 4 times in PBS and imaged on an Eclipse TE300 fluorescent microscope (Nikon).

### MTT assay

Cells were assayed in biological triplicate for viable cell numbers using the CellTiter 96 Non-Radioactive Cell Proliferation Assay (MTT) (Promega) according to the manufacturer’s protocol. Briefly, cells plated in triplicate were treated with dye solution for 2 h and then treated with stop solution and incubated for an additional 1 h. Absorbance at 570 nm was measured with a Synergy plate reader (BioTek). Medium only absorbance was subtracted from all samples, and experimental samples were reported as the average fold change relative to untreated control cells. Statistical comparisons were preformed using two-tailed Student’s *t* tests with a significance cutoff of *p* = 0.05.

### Karyotype

The pluripotent reprogramming required for the derivation of iPSCs can frequently result in copy number variation that would change the developmental potential and malignant capacity of the cells or influence the expression of the integrated bacterial luciferase genes. To ensure these effects were not present in the cells used for this work, live cell line samples were sent to Cell Line Genetics (Madison, WI) for G-band karyotyping according to the vendor’s instructions.

### Doxycycline induction and compound challenge testing

All compounds were sourced from MilliporeSigma and were resuspended in DMSO. Cardiomyocyte toxicity reporting was performed by seeding biological triplicate replicates of 7.5 × 10^4^ 25-day-old cells per well in Matrigel-coated 96-well plates. Cells were challenged 24–48 h after seeding. Challenge compounds were prepared by serial dilution. All tests included vehicle (DMSO only) and unchallenged (medium only) controls. Assays were performed using an IVIS Lumina imaging system (PerkinElmer) at the indicated times. Doxycycline induction experiments used identical cell seeding and monitoring procedures but substituted doxycycline treatment for chemical challenge as indicated. Statistical comparisons were performed using two-tailed Student’s *t* tests with a significance cutoff of *p* = 0.05.

### In vivo cell imaging

Autobioluminescent hADMSCs were prepared for animal injection by first washing once with PBS, then dissociating with Accutase. Dissociated cells were pelleted and washed 3× with PBS. After washing, the indicated number of cells was concentrated into 50 (tail vein) or 100 (intraperitoneal) μL of PBS and injected into triplicate biological replicate FVB/NHsd mice (Envigo). Imaging was performed using an IVIS Lumina imaging system at the indicated times. Statistical comparisons were performed using two-tailed Student’s *t* tests with a significance cutoff of *p* = 0.05.

## Supplementary information

**Additional file 1: Fig. S1.** Luciferin:luciferase component integration and transcriptional expression ratios in autobioluminescent cells following extended time in culture. PDF File detailing the results of qPCR and qRT-PCR experiments to determine luciferin:luciferase integration and expression post-transfection. (a) Genomic DNA from iPSC-*lux* lines 11 passages after stable transfection with a 30:1 molar ratio of Stem-*lux*_CDEF_:Stem-*lux*_AB_ or cardiomyocytes stably transfected with the tetracycline-repressible *lux* operon (TET-*lux*; 1:1 molar ratio), were probed by qPCR to determine the actual gene expression ratios post-transfection. Because the luciferin and luciferase pathway genes were expressed using 2A elements to concatenate each component into a single open reading frame, the second gene of each operon was used for qPCR analysis. As described in [12], this approach provides an average expression level for each operon while accounting for possible reduced expression of the genes distal to the promoter. (b) qRT-PCR analysis reveals that, despite their 27:1 genomic integration ratio, the luciferin pathway is only transcribed at 15:1 relative to the luciferase pathway. Data is available at https://osf.io/h5qzj/ [15].

**Additional file 2: Fig. S2.** iPSC-*lux* cell lines maintain the physiological markers of their wild type counterparts. PDF file showing the evaluation of physiological effects resulting from continuous autobioluminescent expression. Wild type and autobioluminescent iPSCs display similar (a) growth rates, (b) metabolic activity levels, and (c) relative viability when cultured under identical conditions. (d) Wild type iPSCs cultured for approximately 3 months were fixed and immunohistochemically labeled for Nanog, Oct4, and Ssea-4. The red circle at 100× denotes the region shown at 400×. (e) An iPSC line cultured for 11 passages (approximately 3 months) following genomic integration of Stem-*lux*_CDEF_ and Stem-*lux*_AB_ expresses markers of pluripotency similar to wild type. (f) Pluripotency marker expression was also similar in iPSCs stably transfected with the tetracycline-repressible *lux* operon. Both the (g) constitutive and (h) inducible autobioluminescent iPSC cell lines retained a normal 46, XX karyotype. Data is available at https://osf.io/h5qzj/ [15].

**Additional file 3: Fig. S3.** Tetracycline repressible autobioluminescent iPSC cells differentiated into cardiomyocytes and challenged with increasing concentrations of known cardiomodulators. PDF file demonstrating the use of autobioluminescent cardiomyocytes for cardiotoxicity screening. Similar to constitutively autobioluminescent iPSCs and iPSC-derived cardiomyocytes, the cells were capable of reporting changes in viability due to chemical challenge via corresponding changes in autobioluminescent output. Values are representative of *N* = 3 replicates. Error bars represent standard error of the means. p/s/cm^2^/sr; photons/second/cm^2^/steradian. Data is available at https://osf.io/h5qzj/ [15].

**Additional file 4: Fig. S4.** The autobioluminescent phenotype can be introduced into MSCs similarly to iPSCs. PDF file showing the result of transfecting different luciferin:luciferase ratios into MSCs and how the resulting autobioluminescent cells can be used to track population size. (a) Light output of MSCs transfected with increasing ratios of Stem-*lux*_CDEF_:Stem-*lux*_AB_ from 1:1 to 40:1. The ideal 20-30:1 ratio identified for MSCs was the same as that for iPSCs. (b) The autobioluminescent output of MSCs transfected with Stem-*lux*_CDEF_ and Stem-*lux*_AB_ correlated with cell number similar to iPSCs. Values are representative of *N* = 3 replicates. Error bars represent standard error of the means. p/s/cm^2^/sr; photons/second/cm^2^/steradian. Data is available at https://osf.io/h5qzj/ [15].

**Additional file 5: Fig. S5.** In vivo imaging of autobioluminescent hADMSCs. PDF file showing the injection of autobioluminescent MSCs into a small animal model. (a) Increasing numbers of hADMSCs expressing genomically integrated Stem-*lux*_CDEF_ and Stem-*lux*_AB_ were injected intraperitoneally into fvb inbred mice at the locations indicated by the red circles (number of injected cells indicated below red circle) and assayed after 10 min. (b) The resulting autobioluminescent signals showed a strong correlation to injected cell number. p/s/cm^2^/sr; photons/second/cm^2^/steradian. Data is available at https://osf.io/h5qzj/ [15].

**Additional file 6: Fig. S6.** Autobioluminescent hADMSCs show accumulation in the lungs following tail vein injection. PDF file showing the accumulation of autobioluminescent MSCs in the lungs of a small animal model following tail vein injection. 1 × 10^6^ hADMSCs with genomically integrated Stem-*lux*_CDEF_ and Stem-*lux*_AB_ were injected into the tail vein of fvb inbred mice. At 1 h post-injection the subjects were sacrificed and dissected to determine the inter-organellar localization of the labeled cells. p/s/cm^2^/sr; photons/second/cm^2^/steradian.

**Additional file 7: Fig. S7.** Immunohistochemical confirmation of cardiac differentiation. PDF File showing staining of cardiomyocytes with the anti-Troponin-T antibody to confirm successful differentiation. Following the onset of beating, cardiomyocyte differentiation was confirmed by staining with the primary antibody: Troponin T, Cardiac Isoform Ab-1, Mouse Monoclonal Antibody, Clone: 13-11 Isotype: IgG1 and visualizing with the secondary antibody: Goat anti Mouse IgG (H+L) Alexa Fluor 488. Cells were imaged using both the transmitted light and green fluorescent protein (GFP) channels of an EVOS M5000 Cell Imaging System at 40× magnification.

**Additional file 8: Table S1.** Antibodies used in this study. PDF File detailing the antibodies used in this study.

## Data Availability

All data generated or analyzed during this study are included in this published article, its supplementary information files, and available in the Center for Open Science repository, https://osf.io/7mpd5/.
