# Supplementary figures and images for: Real-time tracking of stem cell viability, proliferation, and differentiation with autonomous bioluminescence imaging
Source: BMC Biol. 2020 Jul 3;18:79. doi: 10.1186/s12915-020-00815-2 (PMC7333384; doi:10.1186/s12915-020-00815-2)

a

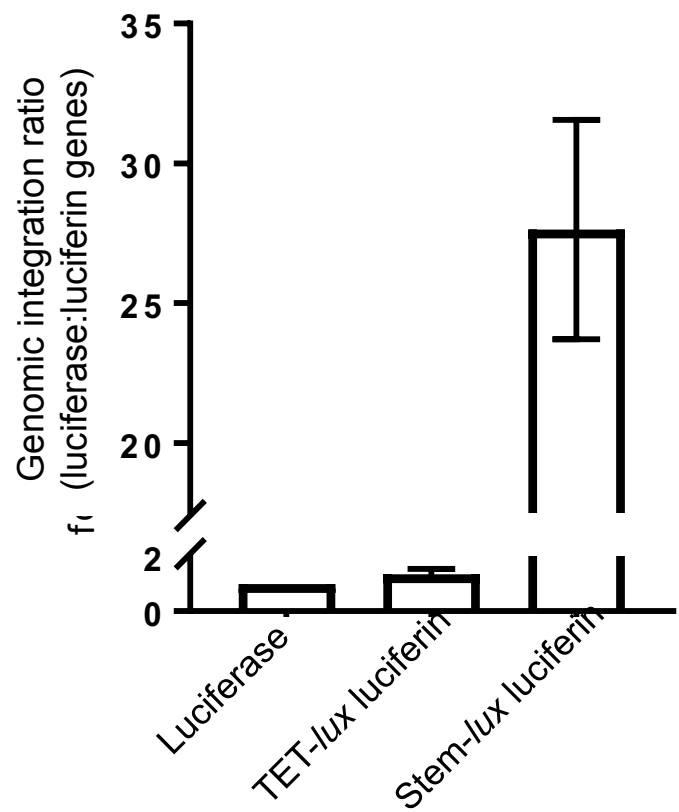

b

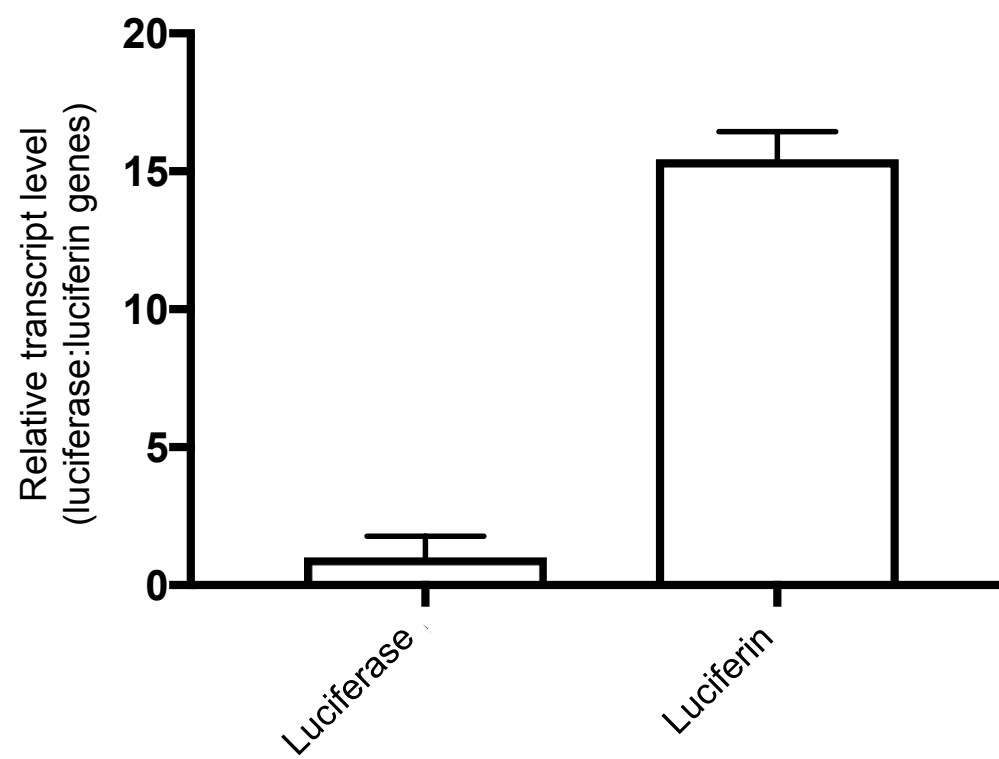

Supplement: Supplementary file 1 — Additional file 1: Fig. S1. Luciferin:luciferase component integration and transcriptional expression ratios in autobioluminescent cells following extended time in culture. PDF File detailing the results of qPCR and qRT-PCR experiments to determine luciferin:luciferase integration and expression post-transfection. (a) Genomic DNA from iPSC-lux lines 11 passages after stable transfection with a 30:1 molar ratio of Stem-luxCDEF:Stem-luxAB or cardiomyocytes stably transfected with the tetracycline-repressible lux operon (TET-lux; 1:1 molar ratio), were probed by qPCR to determine the actual gene expression ratios post-transfection. Because the luciferin and luciferase pathway genes were expressed using 2A elements to concatenate each component into a single open reading frame, the second gene of each operon was used for qPCR analysis. As described in [12], this approach provides an average expression level for each operon while accounting for possible reduced expression of the genes distal to the promoter. (b) qRT-PCR analysis reveals that, despite their 27:1 genomic integration ratio, the luciferin pathway is only transcribed at 15:1 relative to the luciferase pathway. Data is available at https://osf.io/h5qzj/ [15]. [file 12915_2020_815_MOESM1_ESM.pdf]

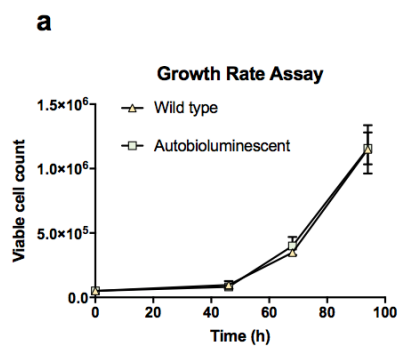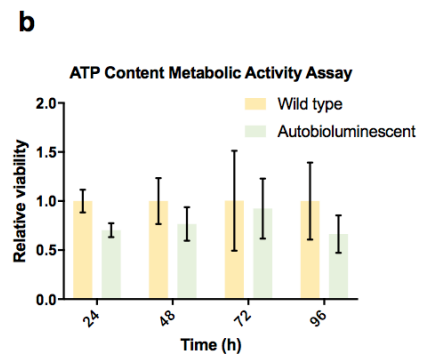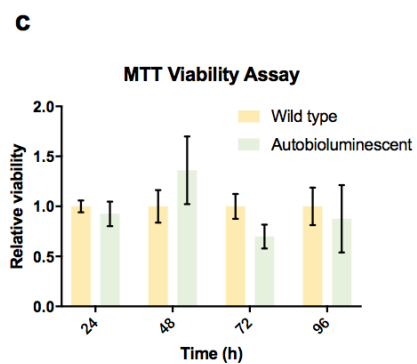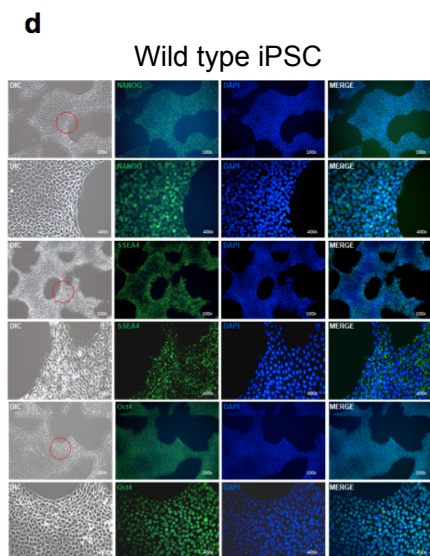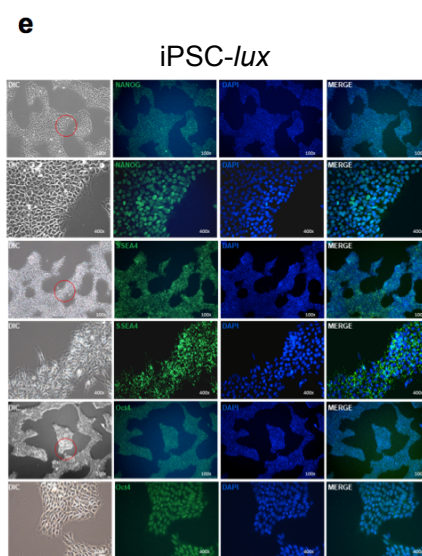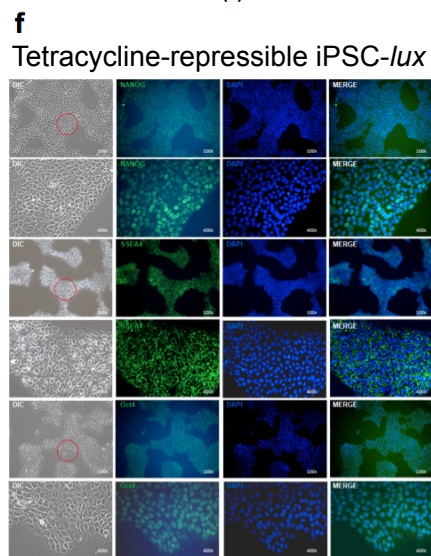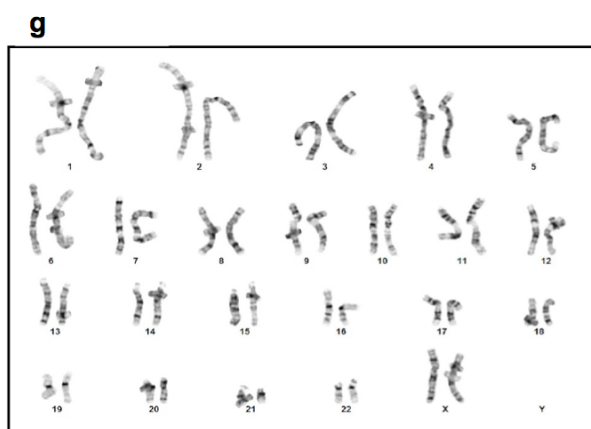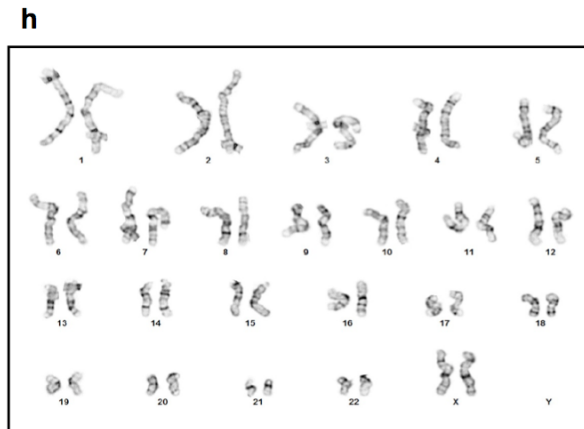

Supplement: Supplementary file 2 — Additional file 2: Fig. S2. iPSC-lux cell lines maintain the physiological markers of their wild type counterparts. PDF file showing the evaluation of physiological effects resulting from continuous autobioluminescent expression. Wild type and autobioluminescent iPSCs display similar (a) growth rates, (b) metabolic activity levels, and (c) relative viability when cultured under identical conditions. (d) Wild type iPSCs cultured for approximately 3 months were fixed and immunohistochemically labeled for Nanog, Oct4, and Ssea-4. The red circle at 100× denotes the region shown at 400×. (e) An iPSC line cultured for 11 passages (approximately 3 months) following genomic integration of Stem-luxCDEF and Stem-luxAB expresses markers of pluripotency similar to wild type. (f) Pluripotency marker expression was also similar in iPSCs stably transfected with the tetracycline-repressible lux operon. Both the (g) constitutive and (h) inducible autobioluminescent iPSC cell lines retained a normal 46, XX karyotype. Data is available at https://osf.io/h5qzj/ [15]. [file 12915_2020_815_MOESM2_ESM.pdf]

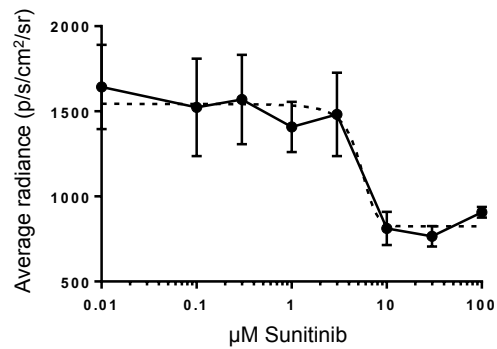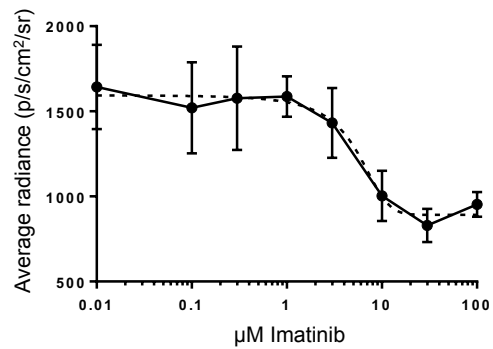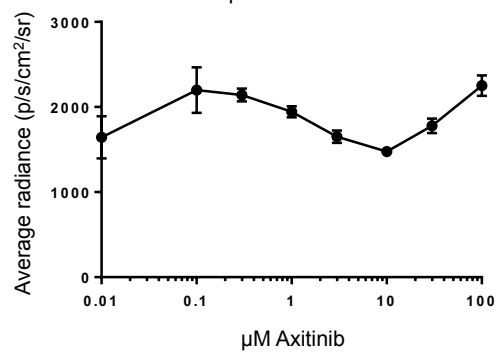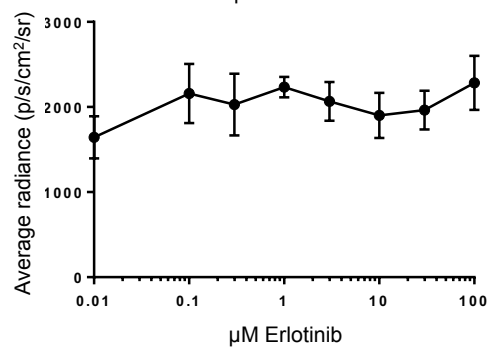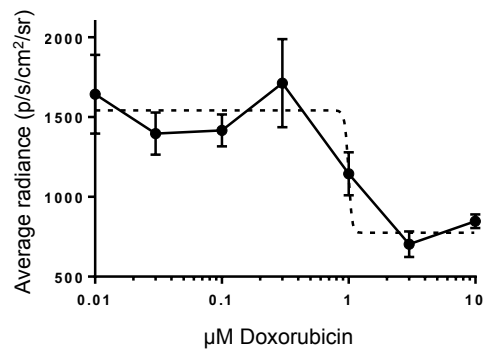

Supplement: Supplementary file 3 — Additional file 3: Fig. S3. Tetracycline repressible autobioluminescent iPSC cells differentiated into cardiomyocytes and challenged with increasing concentrations of known cardiomodulators. PDF file demonstrating the use of autobioluminescent cardiomyocytes for cardiotoxicity screening. Similar to constitutively autobioluminescent iPSCs and iPSC-derived cardiomyocytes, the cells were capable of reporting changes in viability due to chemical challenge via corresponding changes in autobioluminescent output. Values are representative of N = 3 replicates. Error bars represent standard error of the means. p/s/cm2/sr; photons/second/cm2/steradian. Data is available at https://osf.io/h5qzj/ [15]. [file 12915_2020_815_MOESM3_ESM.pdf]

**a**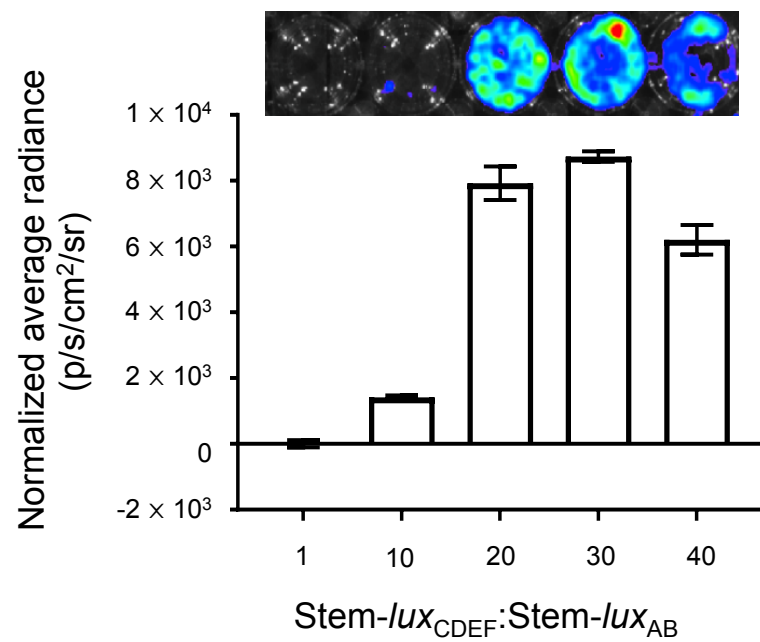**b**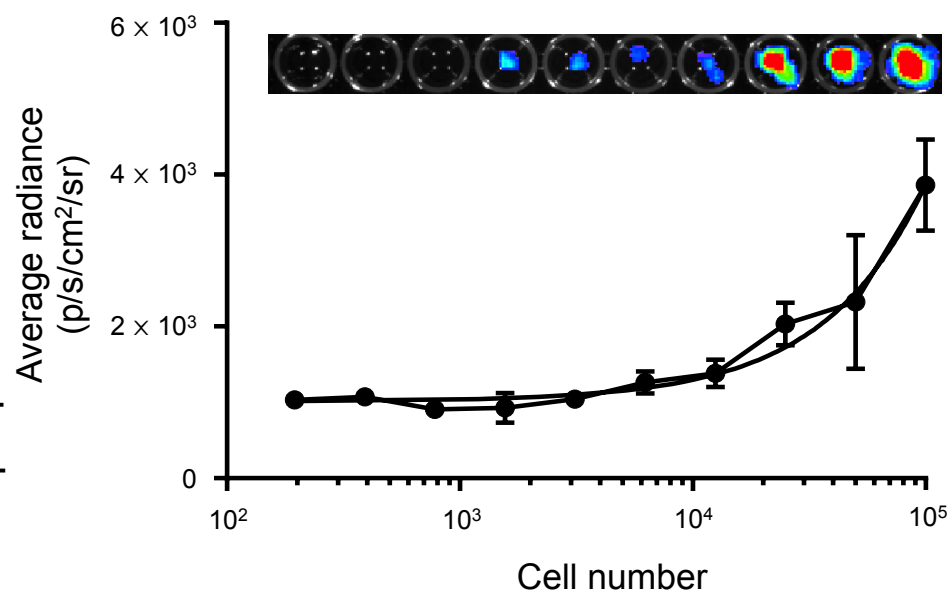

Supplement: Supplementary file 4 — Additional file 4: Fig. S4. The autobioluminescent phenotype can be introduced into MSCs similarly to iPSCs. PDF file showing the result of transfecting different luciferin:luciferase ratios into MSCs and how the resulting autobioluminescent cells can be used to track population size. (a) Light output of MSCs transfected with increasing ratios of Stem-luxCDEF:Stem-luxAB from 1:1 to 40:1. The ideal 20-30:1 ratio identified for MSCs was the same as that for iPSCs. (b) The autobioluminescent output of MSCs transfected with Stem-luxCDEF and Stem-luxAB correlated with cell number similar to iPSCs. Values are representative of N = 3 replicates. Error bars represent standard error of the means. p/s/cm2/sr; photons/second/cm2/steradian. Data is available at https://osf.io/h5qzj/ [15]. [file 12915_2020_815_MOESM4_ESM.pdf]

**a**

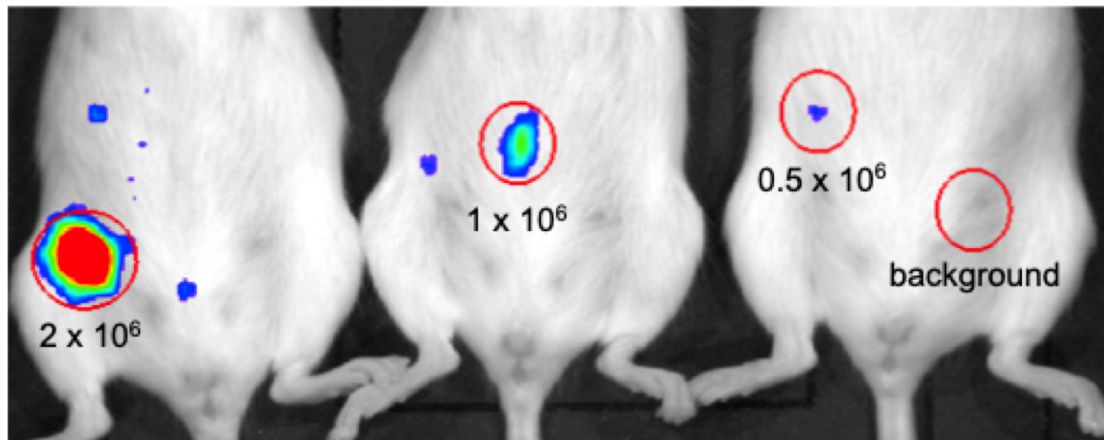

**b**

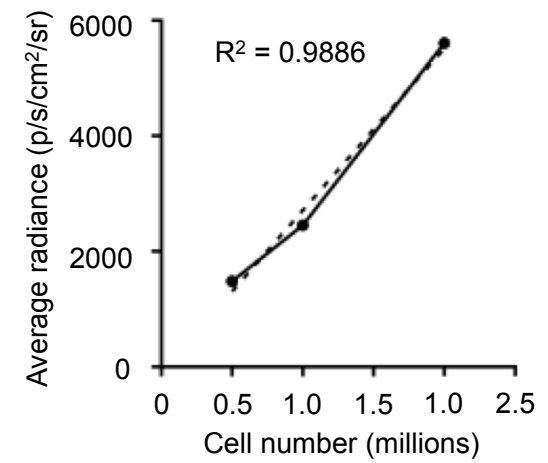

Supplement: Supplementary file 5 — Additional file 5: Fig. S5. In vivo imaging of autobioluminescent hADMSCs. PDF file showing the injection of autobioluminescent MSCs into a small animal model. (a) Increasing numbers of hADMSCs expressing genomically integrated Stem-luxCDEF and Stem-luxAB were injected intraperitoneally into fvb inbred mice at the locations indicated by the red circles (number of injected cells indicated below red circle) and assayed after 10 min. (b) The resulting autobioluminescent signals showed a strong correlation to injected cell number. p/s/cm2/sr; photons/second/cm2/steradian. Data is available at https://osf.io/h5qzj/ [15]. [file 12915_2020_815_MOESM5_ESM.pdf]

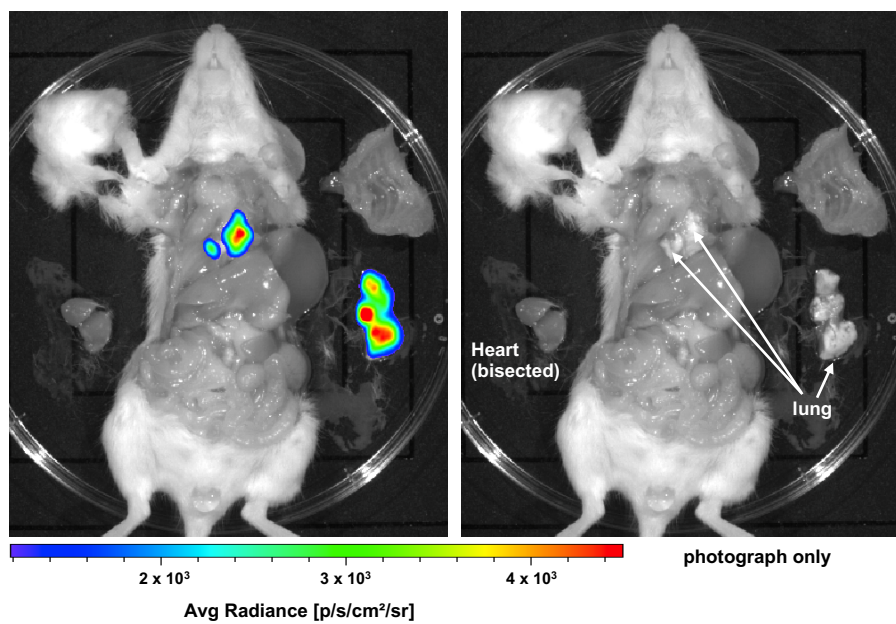

Supplement: Supplementary file 6 — Additional file 6: Fig. S6. Autobioluminescent hADMSCs show accumulation in the lungs following tail vein injection. PDF file showing the accumulation of autobioluminescent MSCs in the lungs of a small animal model following tail vein injection. 1 × 10^6^ hADMSCs with genomically integrated Stem-luxCDEF and Stem-luxAB were injected into the tail vein of fvb inbred mice. At 1 h post-injection the subjects were sacrificed and dissected to determine the inter-organellar localization of the labeled cells. p/s/cm2/sr; photons/second/cm2/steradian. [file 12915_2020_815_MOESM6_ESM.pdf]

Troponin-T (1:500)

+

Goat-anti-Mouse 488 (1:1000)

Goat-anti-Mouse 488 (1:1000)

Transmitted light

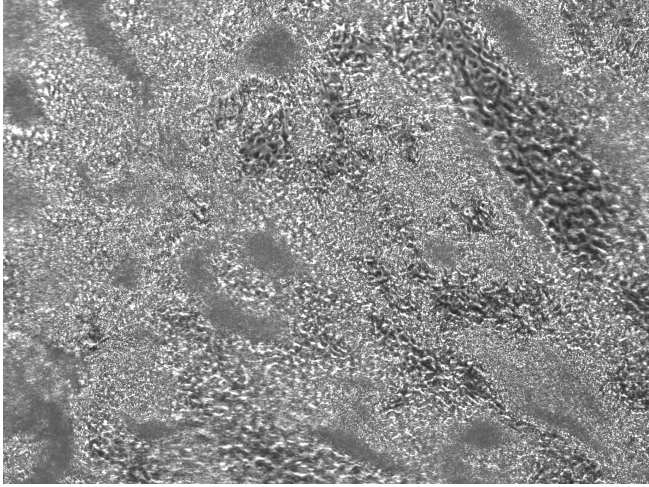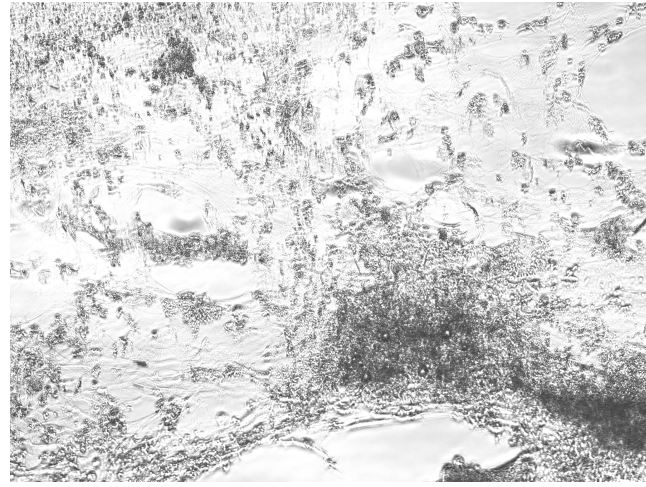

GFP

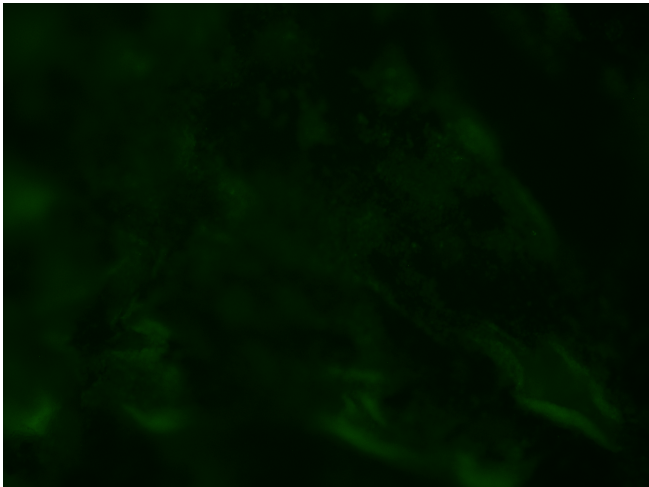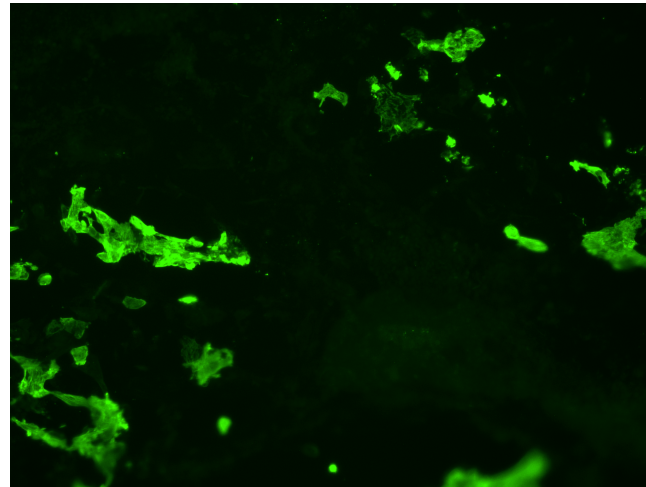

Supplement: Supplementary file 7 — Additional file 7: Fig. S7. Immunohistochemical confirmation of cardiac differentiation. PDF File showing staining of cardiomyocytes with the anti-Troponin-T antibody to confirm successful differentiation. Following the onset of beating, cardiomyocyte differentiation was confirmed by staining with the primary antibody: Troponin T, Cardiac Isoform Ab-1, Mouse Monoclonal Antibody, Clone: 13-11 Isotype: IgG1 and visualizing with the secondary antibody: Goat anti Mouse IgG (H+L) Alexa Fluor 488. Cells were imaged using both the transmitted light and green fluorescent protein (GFP) channels of an EVOS M5000 Cell Imaging System at 40× magnification. [file 12915_2020_815_MOESM7_ESM.pdf]
