# Supplementary material for: Real-time tracking of stem cell viability, proliferation, and differentiation with autonomous bioluminescence imaging
Source: BMC Biol. 2020 Jul 3;18:79. doi: 10.1186/s12915-020-00815-2 (PMC7333384; doi:10.1186/s12915-020-00815-2)
Supplement: Supplementary file 8 — Additional file 8: Table S1. Antibodies used in this study. PDF File detailing the antibodies used in this study. [file 12915_2020_815_MOESM8_ESM.pdf]

Table S1. Antibodies used in this study.

Antibody	Manufacturer	Dilution
SSEA4 Monoclonal Antibody (MC-813-70)	ThermoFisher Scientific<br>Catalog# MA1-021<br>RRID# AB_2536687	1:100
Nanog Monoclonal Antibody (hNanog.2)	ThermoFisher Scientific<br>Catalog# 14-5768-82<br>RRID# AB_467572	1:50
Oct4/Pou5F1 Monoclonal Antibody<br>Clone: 9B7	ThermoFisher Scientific<br>Catalog# MA1-104<br>RRID# AB_2536771	1:50
Goat anti-Mouse IgG (H+L) Cross-Adsorbed Secondary Antibody<br>Alexa Fluor 488	ThermoFisher Scientific<br>Catalog# A-11001<br>RRID# AB_2534069<br>Lot# 2140660	1:500
Troponin T, Cardiac Isoform Ab-1, Mouse Monoclonal Antibody	ThermoFisher Scientific<br>Catalog #MS295P0<br>Lot# 295P1703H	1:500
